# Burg-Aided 2D MIMO Array Extrapolation for Improved Spatial Resolution

**DOI:** 10.3390/s25206310

**Published:** 2025-10-12

**Authors:** Muge Bekar, Ali Bekar, Anum Pirkani, Christopher John Baker, Marina Gashinova

**Affiliations:** 1 Department of Electronic, Electrical and Systems Engineering, University of Birmingham, Birmingham B15 2TT, UK or alibekar@ohu.edu.tr (A.B.); a.a.a.pirkani@bham.ac.uk (A.P.); c.j.baker.1@bham.ac.uk (C.J.B.); m.s.gashinova@bham.ac.uk (M.G.); 2 Department of Electrical and Electronics Engineering, Abdullah Gul University, Kayseri 38080, Türkiye; 3 Department of Electrical and Electronics Engineering, Nigde Omer Halisdemir University, Nigde 51240, Türkiye

**Keywords:** Burg algorithm, 2D MIMO antenna, Burg-aided MIMO, autoregressive method

## Abstract

In this paper, the extrapolation of a 2D multiple-input multiple-output (MIMO) array is proposed using the Burg algorithm to achieve higher angular resolution beyond that of the corresponding 2D MIMO virtual array. The main advantage of such an approach is that it allows us to dramatically decrease both the physical size and the number of antenna elements of the MIMO array. The performance and limitations of the Burg algorithm are examined through both simulation and experimentation at 77 GHz. The experimental methodology used to acquire 3D data of range, azimuth and elevation information with the 1D MIMO off-the-shelf radar is described. Using this method, the performance of the proposed array can be tested experimentally, especially at frequencies where it is desired to assess the antenna response prior to fabricating the antenna.

## 1. Introduction

Radar applications can achieve high range resolution thanks to the high available bandwidth at mm wavelengths. In addition to high range resolution, most modern radar applications require high angular resolution in both the azimuth and elevation dimensions in order to resolve closely located targets within the radar field of view. The angular resolution, for a given transmission frequency, is defined by antenna effective aperture size, and in the case of a MIMO array, the same angular resolution can be achieved with significantly fewer antenna elements, compared with a fully populated phased array with half-wavelength element spacing [1,2]. Nonetheless, both the total number of antenna elements in a MIMO configuration and the size of the antenna can still be large when high angular resolutions are required, which would limit its suitability for applications where the space available for sensors is limited, such as automotive applications. Additionally, when transmitters in MIMO are activated at different time slots in time division multiplexing (TDM) mode, the radar response time increases depending on the number of transmitters, which causes an unwanted reduction in Doppler unambiguous range, critical in MIMO-SAR applications [3]. Therefore, further reducing the total number of antenna elements, especially in the number of transmitters, is a desirable approach to reduce the cost, size and weight but also to decrease the radar response time in TDM MIMO.

The angular resolution and sensitivity of a conventional MIMO antenna is defined by an array factor expressed as a sinc function with approximately −13 dB for the highest sidelobe level without applying weightings [4]. When the number of antenna elements in MIMO is reduced by retaining the edge elements and repositioning the inner elements within the array, the same angular resolutions are achieved, whereas grating lobes and high sidelobe levels which limit the performance of radar detection may occur. In such contexts, finding the antenna positions in thinned MIMO to achieve the required angular resolution with acceptable sidelobe levels represents an optimization problem [5]. An exhaustive search can be performed when there are few potential solutions for the available positions of the antenna elements. However, it is nearly impossible to look through all potential solutions when the search space is large. Several optimization techniques are reported in the literature, such as simulated annealing, the genetic algorithm, particle swarm optimization and ant colony optimization, for finding suitable configurations of antenna element positions to design thinned linear, planar and MIMO antennas [6,7,8,9,10,11,12]. All such techniques require a very high number of iterations. Moreover, the achieved antenna pattern may give irregular sidelobe levels in the background, which can limit target detection.

Another approach to achieve higher angular resolution is to use super-resolution techniques such as the multiple signal classification (MUSIC) algorithm [13], but this method requires multiple snapshots of the scene as well as prior knowledge of the number of targets within the scene. Also, radar data can be extrapolated through the matrix pencil method [14] and machine learning [15,16], improving the resolution without expanding the physical antenna aperture or using multiple snapshots. However, the number of singular values in the matrix pencil method must be chosen carefully to avoid having false targets, whereas machine learning techniques require a large number of training data and are computationally expensive. To extrapolate radar data, autoregressive (AR) methods can be employed [17,18,19,20]. Several methods are reported to predict the coefficients of the AR model such as Yule–Walker, the Burg algorithm (BA) and the covariance method. In [20], the performance of these AR methods has been compared, and it is stated that the BA has a better performance in terms of the image quality in a noisy environment and makes more reliable and stable estimations compared to other methods [21,22].

The performance of the BA has been well studied and presented in the literature. For instance, in [23], the missing data in synthetic aperture radar (SAR) imaging were predicted using the BA, and the results demonstrated a significant improvement in image quality. In [24], the BA was used with the Doppler Beam Sharpening (DBS) technique for side-looking radar geometry to enhance cross-range resolution. The BA was also used in a 1D MIMO concept to both interpolate antenna element positions where data were missing and to extrapolate where super-resolution was needed in [25]. In [26], the BA has been combined with the MIMO-DBS technique in order to increase angular resolution in the forward-looking direction and to achieve further enhanced angular resolution in the lateral directions. To the best of our knowledge, previously, the Burg algorithm has been applied only to 1D arrays, and 2D extrapolation has been initially presented in our recent conference paper [27].

In this study, the Burg algorithm is applied to extrapolate and/or interpolate the VA elements so that high resolution in both azimuth and elevation is achieved, retaining the initial compact 2D MIMO array in a fast and easy to implement approach, allowing us to retrieve the target’s positional information without the requirement of additional input from the user or the use of optimization techniques whilst giving a stable prediction filter [28,29,30,31].

To achieve higher angular resolution beyond that of the equivalent MIMO virtual array (VA), the virtual array elements are extrapolated towards both azimuth and elevation directions by applying the autoregressive method. In this way, it is shown that the size and number of elements of MIMO physical arrays can be significantly reduced.

Additionally, this approach can address an issue of missing experimental data due to manufacturing or experimental failure. Instead of re-manufacturing the antenna and repeating the experiment, the missing data can be estimated through the proposed method here.

The performance of the reconstructed MIMO array using the Burg algorithm is demonstrated here in both 1D and 2D configurations, and the limitations of the algorithm are explained in detail. The validation of the 1D MIMO concept as in [25] is presented in this paper for the sake of ensuring the integrity of the material, alongside new analyses and results. Validation and analysis of the proposed approach to enhancing the performance of the 2D MIMO array is made through both simulation and experiments at 77 GHz. The experimental methodology used to acquire 3D data (range, azimuth and elevation information) using a 1D MIMO radar is described. Using this approach, the antenna configuration can be tested experimentally using off-the-shelf 1D MIMO chipset where it is desired to observe the antenna response prior to fabricating the antenna.

The current paper builds on the work presented in the conference paper [27]. Specifically, the contributions of this paper are as follows: the introduction of a rule-based 2D design procedure for a compact MIMO system capable of (1) algorithm-based interpolation and extrapolation of regular and sparsely positioned elements; (2) the demonstration of several design layouts and evaluation of their performance; (3) the introduction of a detailed experimental approach to acquiring 2D MIMO data using a 1D MIMO array. This paper provides significantly extended validation results through both simulation and experimentation at 77 GHz.

It is noted that throughout this article, the MIMO array, the virtual array of the MIMO array and the Burg-aided MIMO array are called physical array, virtual array and extrapolated array, respectively.

The rest of this paper is organized as follows. Section 2 gives a brief mathematical explanation of both 1D and 2D MIMO operational principles to define the notation used in the paper. Section 3 explains the Burg algorithm in detail, whereas the proposed Burg-aided MIMO method is described in Section 4. Simulation and experimental results are presented and discussed in Section 5 and 6, respectively. Conclusions are formulated and further steps are outlined in Section 7.

## 2. MIMO Antenna

For the integrity of this paper, this section provides a brief mathematical description of the beamforming principle of both 1D and 2D MIMO arrays.

### 2.1. 1D MIMO

Assuming a 1D MIMO array consists of $N_{T}$ transmit and $N_{R}$ receive antenna elements aligned along the *x*-axis, its transmit and receive steering vectors, $a_{1-D}\left(\theta \right)$ and $b_{1-D}\left(\theta \right),$ respectively, for a target positioned in the far field at an azimuth angle θ from the boresight, are given by

(1) $$a_{1-D}\left(\theta \right)={\left[e^{{-jkx}_{T_{1}}sin\theta }\;e^{-{jkx}_{T_{2}}sin\theta }\;\cdots {\;e}^{{-jkx}_{T_{N_{T}}}sin\theta }\right]}^{T}$$

and

(2) $$b_{1-D}\left(\theta \right)={\left[e^{{-jkx}_{R_{1}}sin\theta }\;e^{{-jkx}_{R_{2}}sin\theta }\;\cdots {\;e}^{{-jkx}_{R_{N_{R}}}sin\theta }\right]}^{T},$$

where k=2π/λ is the wavenumber, λ is the wavelength, and $x_{T_{n}}$ and $x_{R_{n}}\;$are *x*-coordinates of the n^th^ transmit and receive antenna elements, respectively.

The steering vector of the whole 1D virtual array is defined as a Kronecker product of transmit and receive steering vectors, and it can be written as in (3):

(3) $$v_{1-D}\left(\theta \right)={\left(\begin{array}{c} e^{\begin{array}{c} -jk(x_{T_{1}}+x_{R_{1}})\sin\theta \end{array}}\\ e^{\begin{array}{c} -jk(x_{T_{1}}+x_{R_{2}})\sin\theta \end{array}}\\ \vdots \\ e^{\begin{array}{c} -jk(x_{T_{1}}+x_{R_{N_{R}}})\sin\theta \end{array}}\\ \vdots \\ e^{\begin{array}{c} -jk(x_{T_{N_{T}}}+x_{R_{N_{R}}})\sin\theta \end{array}} \end{array}\right)}_{N_{T}N_{R}x1}$$

The positions of the virtual array elements are given by the following:

(4) $$l_{1-D}={\left(\begin{array}{c} x_{T_{1}}+x_{R_{1}}\\ x_{T_{1}}+x_{R_{2}}\\ \vdots \\ x_{T_{1}}+x_{R_{N_{R}\;}}\\ \vdots \\ x_{T_{N_{T}}}+x_{R_{N_{R}}} \end{array}\right)}_{N_{T}N_{R}x1}$$

The effective dimension of the virtual array of the 1D MIMO is calculated as

(5) $$l_{x}=\max\left(l_{1-D}\right)-\min\left(l_{1-D}\right).$$

The angular resolution defined at the −3 dB points of the MIMO beamwidth is defined by the effective length, *L*, of the virtual array, $\theta_{3dB}≅0.88\lambda /L$, and, in the case of 1D MIMO, by the azimuth angular resolution, $l_{x}$.

### 2.2. 2D MIMO

**Figure 1:**
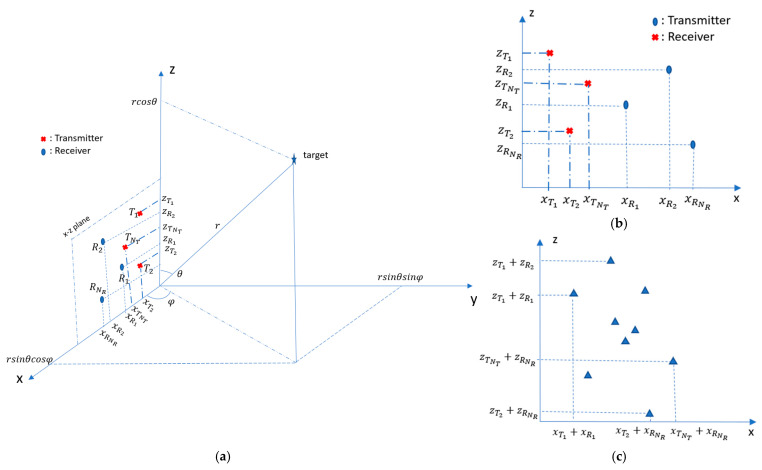
(**a**) 2D MIMO in *xz*-plane presented in spherical coordinate system; (**b**) example of 2D MIMO antenna configuration; (**c**) virtual array of (**b**) [12].

In 2D MIMO, the antenna elements might be placed along two orthogonal axes, for instance, aligned along horizontal, *x*, and vertical, *z*, axes in order to obtain the required spatial resolutions in both azimuth and elevation. Assuming that the target is in the far field of the antenna, then for all elements of the antenna, the angular directions to the target can be assumed to be the same for the colocated MIMO array. If the target is positioned at the point with the coordinate (r,φ,θ), where r is the range, φ is the azimuth angle and θ is the elevation angle with regard to the MIMO array origin as shown in Figure 1, then for each pair of transmit and receive elements, the path lengths and, therefore, the phase of the signal arriving from the target differ, thus giving rise to a virtual element.

The steering vector of the corresponding virtual array is the Kronecker product of the transmit and receive steering vectors, and can be written as follows [12]:

(6) $$g\left(\varphi ,\theta \right)={\left(\begin{array}{c} e^{\begin{array}{c} -jk((x_{T,1}+x_{R,1})\sin\theta cos\varphi +(z_{T,1}+z_{R,1})cos\theta ) \end{array}}\\ e^{\begin{array}{c} -jk((x_{T,1}+x_{R,2})\sin\theta cos\varphi +(z_{T,1}+z_{R,2})cos\theta ) \end{array}}\\ \vdots \\ e^{\begin{array}{c} -jk((x_{T,1}+x_{{R,N}_{R}})\sin\theta cos\varphi +(z_{T,1}+z_{{R,N}_{R}})cos\theta ) \end{array}}\\ \vdots \\ e^{\begin{array}{c} -jk((x_{T,N_{T}}+x_{R,1})\sin\theta cos\varphi +(z_{{T,N}_{T}}+z_{R,1})cos\theta ) \end{array}}\\ \vdots \\ e^{\begin{array}{c} -jk((x_{T,N_{T}}+x_{R,N_{R}})\sin\theta cos\varphi +(z_{T,N_{T}}+z_{R,N_{R}})cos\theta ) \end{array}} \end{array}\right)}_{N_{T}N_{R}x1}$$

where $\left(x_{\left\{T,n\right\}},z_{\left\{T,n\right\}}\right){|}_{\left\{n=1\ldots N_{T}\right\}}$ and $\left(x_{\left\{R,m\right\}},z_{\left\{R,m\right\}}\right){|}_{\left\{m=1\ldots N_{R}\right\}}\;$ are coordinates of the phase center of the *n*-th transmit and *m*-th receive antenna elements, respectively.

The coordinates of virtual antenna elements are

(7) $$v_{x}={\left(\begin{array}{c} x_{T,1}+x_{R,1}\\ x_{T,1}+x_{R,2}\\ \vdots \\ x_{T,N_{T}}+x_{R,N_{R}} \end{array}\right)}_{N_{T}N_{R}x1},\;\;\;\;\;\;\;v_{z}={\left(\begin{array}{c} z_{T,1}+z_{R,1}\\ z_{T,1}+z_{R,2}\\ \vdots \\ z_{T,N_{T}}+z_{R,N_{R}} \end{array}\right)}_{N_{T}N_{R}x1},$$

and the effective dimensions of virtual aperture in the *x*- and *z*-directions, defining the azimuth and elevation angular resolutions, are as follows:

(8) $$\;\;\;\;l_{x}=\max\left(v_{x}\right)-\min\left(v_{x}\right)\;,\;\;\;l_{z}=\max\left(v_{z}\right)-\min\left(v_{z}\right)$$

Physical MIMO elements can be arranged in L-shaped or T-shaped configurations to produce a rectangular VA as in [32,33], where transmit and receive elements are positioned separately along orthogonal axes. Another approach is to place the Tx and Rx antennas at the edges of a rectangle as in [34,35]. A rectangular VA is the most compact form, resulting in a smaller area; therefore, in this paper, a rectangular VA is formed with two different approaches, which will both be used in Section 5.2 with examples of several designs.

**1st approach:** Assuming *xz*- is the plane of the 2D MIMO array, both transmit and receive sub-arrays are also two-dimensional, with $N_{R_{x}}\times \;N_{R_{z}}$ receive and $N_{T_{x}}\times \;N_{T_{z}}$ transmit elements, respectively. The positions of each receive and transmit element on the *x*- and *z*-axes can be formulated as follows:

(9) $$\;\;\;\;Rx_{pos_{w,t}}=\left[\left(w-1\right)dx;\;\left(t-1\right)dz\right]$$

and

(10) $$Tx_{pos_{e,s}}=\left[\left(e-1\right)N_{R_{x}}dx;\;\left(s-1\right)N_{R_{z}}dz\right]$$

where *dx* and *dz* are the spacing between the receive elements on the *x*- and *z*-axes, respectively, $w=1,2,\cdots ,\;N_{R_{x}}$, $t=1,2,\cdots ,\;N_{R_{z}}$, $e=1,2,\cdots ,\;N_{T_{x}}$, and $s=1,2,\cdots ,\;N_{T_{Z}}$.

This antenna configuration gives us a uniform rectangular VA with *dx* and *dz* spacings along the *x*- and *z*-axes, respectively, without producing overlapped virtual elements. When the ±90° unambiguous field of view is needed in both azimuth and elevation directions, the spacing between the receive antenna elements can be chosen as dx=dz=λ/2.

**2nd approach:** Under the same assumptions as above and the positions of each receive element as in (9), the position of the transmit element can be determined by the following equation:

(11) $$Tx_{pos_{e,s}}=\left[1.5\left(e-1\right)dx;\;1.5\left(s-1\right)dz\right]$$

In such case, a uniform rectangular array with spacings of dx/2 and dz/2 on the *x*- and *z*-axes, respectively, is obtained within the inner area of the VA. However, this 2D MIMO arrangement generates sparse elements, with dx and dz spacing on the *x*- and *z*-axes at the edges of the VA. Therefore, to obtain λ/2 spacing between the elements of the inner array of the VA in this configuration, the spacings among receivers and transmitters are λ and 1.5λ, respectively.

It is worth stressing here that following this procedure, as can be seen from either (9) and (10) or (9) and (11), the first Rx and Tx physical elements are co-located at the origin [0;0]. One possible solution would be a design of such elements as a transceiver, though a more straightforward solution would be the separation of the transmitter and receiver areas, as will be demonstrated in Section 5.2. The latter will apparently increase the physical dimension at least in one direction, however retaining the radar’s performance as in the more elegant case of co-located transmitter/receiver elements.

## 3. The Burg Algorithm

For target detection by the phased array, based on the evolution of phases of the actual signals into the receive elements, it is possible to predict the phases of signals for extrapolated, or reconstructed, receive elements, and the problem should actually include the estimation of boundaries of applicability beyond which the prediction is no longer accurate. Autoregressive methods can predict, or extrapolate, future values of a sequence of {u} by examining past values, and the AR model can be defined as [22]

(12) $$u\left[p\right]=-\sum_{q=1}^{M}a_{q}u\left[p-q\right],$$

where M is the model order, $a_{q}$ is the qth model coefficient, $p=M+1,\cdots ,N_{x}$, and $N_{x}$ is the total number of elements in the sequence after extrapolation is performed.

The Burg algorithm can be used to estimate the values of coefficients, $a_{q}$, of the AR model or, in other words, the AR filter. The same algorithm can also be used for interpolation. In radar applications, this will be the case when experimental data are missing, e.g., due to antenna manufacturing or experimental failures, so that the antenna pattern can still be reconstructed. The principle of the iterative-based BA operation is to minimize the combined estimation error, $E_{i}$, through forward and backward prediction:

(13) $$E_{i}={f_{i}}^{H}f_{i}+{b_{i}}^{H}b_{i}$$

where *i* is the iteration number, ${\left(\cdot \right)}^{H}$ is the Hermitian transposition, and $f_{i}$ and $b_{i}$ are forward and backward prediction error vectors, respectively.

The algorithm steps to compute M model, or filter coefficients, whose maximum number is limited by the number of available virtual antenna elements, consists of the following [25,27,36]:

1. At the first iteration (i=0), the initial forward and backward prediction error vectors ($f_{0}^{\prime }$ and $b_{0}^{\prime }$) are assumed to be equal to u:
  (14) $$u={\left[u_{0},\;{\;u}_{1},\;{\;u}_{2},\cdots \;u_{N-1}\right]}^{T}$$

where $u_{n}$ denotes the input signal coming from the $n^{th}$ element (0≤n≤N−1), and the first finite impulse response (FIR) filter coefficient, $a_{0}$, is equal to 1.

2. The first element is then removed from $f_{i}^{\prime }$, and the last element is removed from $b_{i}^{\prime }$ in each iteration so that
  (15) $$f_{i}=f_{i}^{\prime }\left(1:N-i-1\right)\;\;\;\&\;\;b_{i}=b_{i}^{\prime }\left(0:N-i-2\right).$$
3. At each next iteration i, the reflection coefficient ($k_{i}$) is calculated as
  (16) $$k_{i}=-\frac{2{b_{i}}^{H}f_{i}}{{f_{i}}^{H}f_{i}+{b_{i}}^{H}b_{i}}.$$
4. The updated prediction vectors are calculated as
  (17) $$f_{i+1}^{\prime }=f_{i}+{k_{i}b}_{i}\;\;\;\;\&\;\;\;\;b_{i+1}^{\prime }=b_{i}+{{k_{i}}^{*}f}_{i},$$

where ${\left(\cdot \right)}^{*}$ denotes a complex conjugate.

5. The updated FIR coefficients are calculated as
  (18) ai+1=ai0+kiYai∗0  ,  Y=0  ⋯    0    1 ⋮          1     00                 ⋮1   0   ⋯    0,          

where Y is the square antidiagonal matrix of size (i+1)×(i+1). The iterations are repeated (steps 2 to 5) until the number of iterations reached is M.

## 4. Burg-Aided MIMO

In this section, the proposed method to achieve higher angular resolution beyond that of the virtual array of MIMO by applying the BA is described. It requires a uniform array configuration to extrapolate and/or interpolate the array aperture. The spacing between the virtual elements can be any multiples of wavelength such as 0.5λ, λ, or 2λ, but regular spacing between elements is required, though the spacing between the virtual elements along two orthogonal dimensions could be different. These spacings will define the unambiguous resolvable angular field of view, but they do not limit the performance of the BA. It is also worth mentioning that the number of virtual elements along both dimensions does not need to be equal and that the algorithm gives a more accurate estimation with the increase in the number of virtual array elements. Importantly, if the VA is sparse, interpolation must be performed first to fill the missing data of the sparse elements in the VA and make the extrapolation possible.

**Figure 2:**
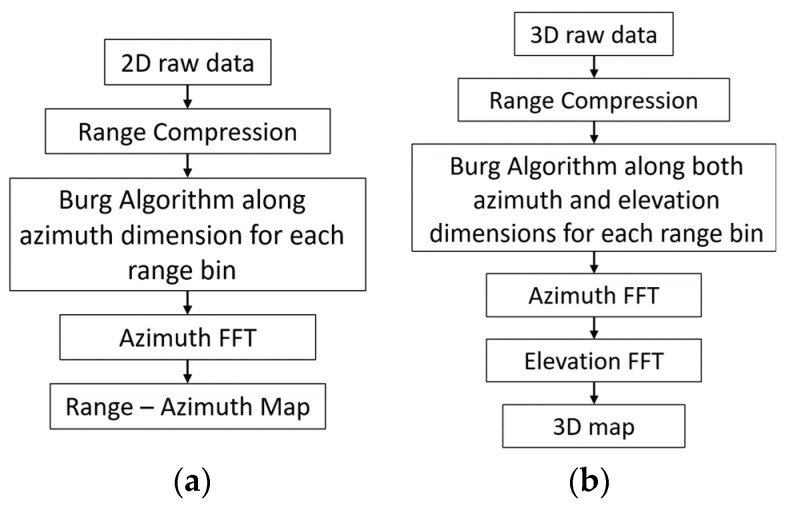
Processing chain of Burg-aided 1D MIMO (**a**) and 2D MIMO (**b**).

A block diagram of the processing chain is shown in Figure 2. To reconstruct the virtual elements of MIMO by interpolating or extrapolating the radar data using the BA, initially range compression is performed on the acquired data in the case of the FMCW signal. Then, for each range bin, the BA steps described in Section 3 are performed along the angular dimension. For clarity, the u in (14) consists of the virtual array data of the related range bin, and the filter coefficients in (12) are found as in steps 1–5, followed by data extrapolation/interpolation through the angular dimension calculation in (12). In the case of 2D MIMO, this must be carried out along both azimuth and elevation dimensions. As a final step, angular Fast Fourier Transform (FFT) is employed to obtain either the range–azimuth map (1D MIMO) or a 3D map (range, azimuth, and elevation) in 2D MIMO. The Burg-aided MIMO steps for both 1D and 2D MIMO arrays are shown in Figure 2.

The computational complexity of Burg-aided 1D MIMO can be estimated as follows:

- $O\left(N_{f}N_{a}\log\left(N_{f}\right)\right)$ for range compression.
- $O\left(N_{f}p_{a}(\varepsilon_{a}N_{a})\log\left(\varepsilon_{a}N_{a}\right)\right)\;$for extrapolation along the azimuth and the azimuth FFT.

Here, $N_{f}$ is the range samples, $N_{a}$ is the sample number of the VA along the azimuth direction, $\varepsilon_{a}$ is the extrapolation factor along the azimuth and $p_{a}$ is the order of the filter in (12) for extrapolating the data along the azimuth dimension.

Hence, the total computation time of BA 1D MIMO is $O\left(N_{f}N_{a}\log\left(N_{f}\right)\right)$ $+O\left(N_{f}p_{a}(\varepsilon_{a}N_{a})\log\left(\varepsilon_{a}N_{a}\right)\right)$.

Regarding the computational complexity of the Burg-aided 2D MIMO, assuming $p_{e}$ is the order of the filter in (12) for extrapolating the data along the elevation dimension, $N_{e}$ is the sample number of the VA along the elevation direction and $\varepsilon_{e}$ is the extrapolation factor along the elevation dimension, the number of operations is as follows:

- $O\left(N_{f}N_{a}N_{e}\log\left(N_{f}\right)\right)$ for range compression.
- $O\left(N_{f}N_{e}p_{a}(\varepsilon_{a}N_{a})\log\left(\varepsilon_{a}N_{a}\right)\right)\;$for extrapolation along the azimuth and the azimuth FFT.
- $O\left(N_{f}\left(\varepsilon_{a}N_{a}\right)p_{e}(\varepsilon_{e}N_{e})\log\left(\varepsilon_{e}N_{e}\right)\right)$ for extrapolation along elevation and the elevation FFT.

Hence, the total computation time of BA 2D MIMO is $O\left(N_{f}N_{a}N_{e}\log\left(N_{f}\right)\right)+O\left(N_{f}N_{e}p_{a}(\varepsilon_{a}N_{a})\log\left(\varepsilon_{a}N_{a}\right)\right)+O\left(N_{f}\left(\varepsilon_{a}N_{a}\right)p_{e}(\varepsilon_{e}N_{e})\log\left(\varepsilon_{e}N_{e}\right)\right)$.

## 5. Simulation Results

In this section, the performance of the Burg algorithm in interpolating and/or extrapolating the available data is analyzed. The performance of the BA with relation to the signal-to-noise ratio (SNR) has been discussed by authors in detail in [37]. It has been shown that at low SNR values (below 15 dB, measured at the output after including processing gains), the algorithm’s performance is not reliable enough to resolve closely spaced targets. With a 15 dB SNR, it begins to achieve reliable separation. At higher output SNR levels, typically in the 20–30 dB range, the probability of correctly resolving closely spaced targets exceeds 90%. In this work, considering typical use cases of automotive radar, we assume that the SNR is sufficiently high to allow for the use of the BA. The operational frequency used in the simulation is 77 GHz with a bandwidth of 2 GHz, which corresponds to experimental results later in this paper.

### 5.1. Burg-Aided 1D MIMO

#### 5.1.1. Estimation of Missing Data (Interpolation)

**Figure 3:**
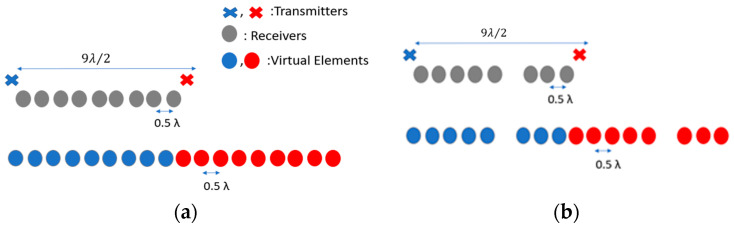
(**a**) MIMO antenna configuration and its positions of VA elements; (**b**) the positions of the VA elements when the 6th receiver is missing/failed.

**Figure 4:**
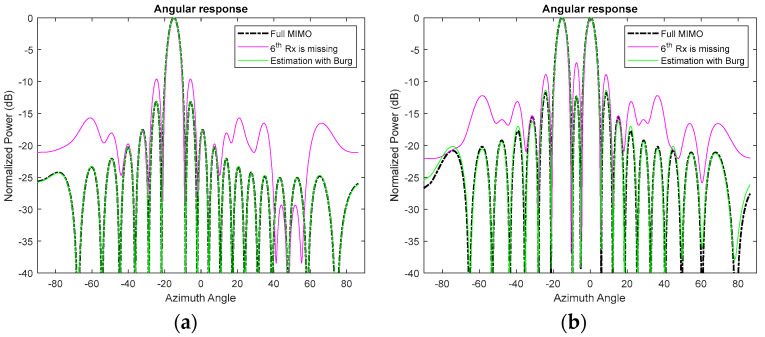
The azimuth cut at a 15 m range in the case of (**a**) a single target at a −15° azimuth angle and (**b**) two targets at −15° and 0° azimuth angles.

While the principle described above is generic and applicable for an arbitrary number of transmit and receive elements, for simplicity of analysis here, we use a conventional MIMO radar comprising two transmitters and nine receivers with a spacing between the transmitters and the receivers of 9λ/2 and λ/2, respectively, as shown in Figure 3a. The corresponding VA consists of 18 uniformly distributed linear elements with a λ/2 spacing, which provides an approximately 6° angular resolution in the direction along array extent. If one of the physical elements of the MIMO array is missing, owing to a hardware failure or experimental data being corrupted, the MIMO can be considered a thinned MIMO. For instance, if the 6th physical receiver element is absent, as shown in Figure 3b, the VA will miss the 6th and 15th virtual elements, which would result in the degradation of performance in terms of the sidelobes of the array pattern, as demonstrated in Figure 4a,b for a single target and two targets, respectively. The angular resolution is not deteriorated as the extreme edge elements, and thus the dimension of the array, are retained. After interpolation with the BA, the response is an exact match to that of the fully populated MIMO array (Figure 4a,b, green lines).

#### 5.1.2. Estimation of Forward Data (Extrapolation)

This section evaluates the performance of the Burg algorithm in extrapolating 1D MIMO radar data, and the limitation to which an extrapolation is expedient is analyzed and discussed with supporting simulation results.

The ability to distinguish targets at the same range but at different angular positions depends on wavelength and effective antenna aperture length. When the Burg algorithm is applied to extrapolate the number of uniformly spaced virtual array elements, the angular resolution can be estimated as follows:

(19) $$\varphi_{3dB_{Burg}}≅\;\frac{0.88\lambda }{\left(\varepsilon_{f}N_{VA}-1\right)d_{VA}}\frac{180}{\pi }\;\;\;\;\left(in\;degrees\right)\;\;\;\;\;\;\;\;\;\;$$

where $\varepsilon_{f}$ is the extrapolation factor, $N_{VA}$ is the number of virtual elements corresponding to the actual MIMO array and $d_{VA}$ is the spacing between the virtual elements.

**Figure 5:**
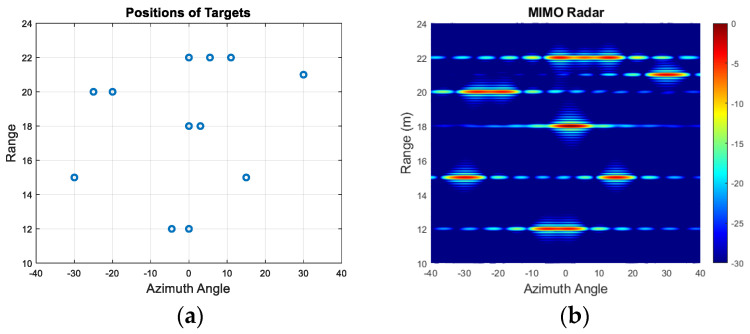
(**a**) The positions of multiple targets; (**b**) the range–azimuth response of 2 Tx and 9 Rx MIMO.

In this subsection, we will demonstrate the performance of extrapolation by the BA with an example of multiple targets (12 targets) which are placed at various azimuth angles and ranges. The simulated scenario is shown in Figure 5a, where blue circles indicate the positions of targets. Targets are located at ranges of [12, 12, 15, 15, 18, 18, 20, 20, 21, 22, 22, 22] m and at azimuth angles of [0°, −4.5°, 15°, −30°, 0°, 3°, −20°, −25°, 30°, 0°, 5.5°, 11°].

**Figure 6:**
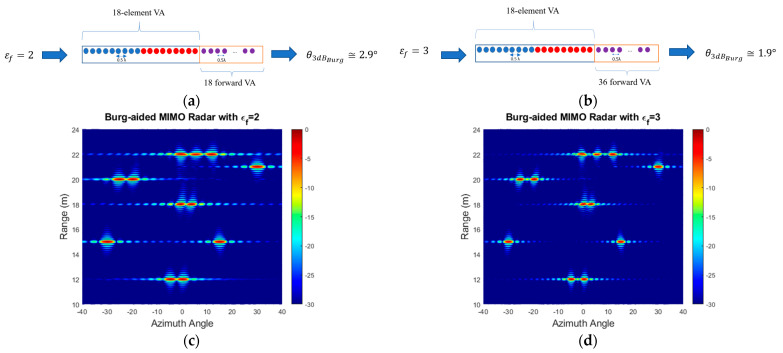
(**a**) The extrapolated VA element and its angular resolution for 2 Tx and 9 Rx based on $\varepsilon_{f}$ is 2, (**b**) the extrapolated VA element and its angular resolution for 2 Tx and 9 Rx based on $\varepsilon_{f}$ is 3, (**c**) the range–azimuth angle response of Burg-aided MIMO with an $\varepsilon_{f}$ of 2 and (**d**) the range–azimuth angle response of Burg-aided MIMO with an $\varepsilon_{f}$ of 2.

**Figure 7:**
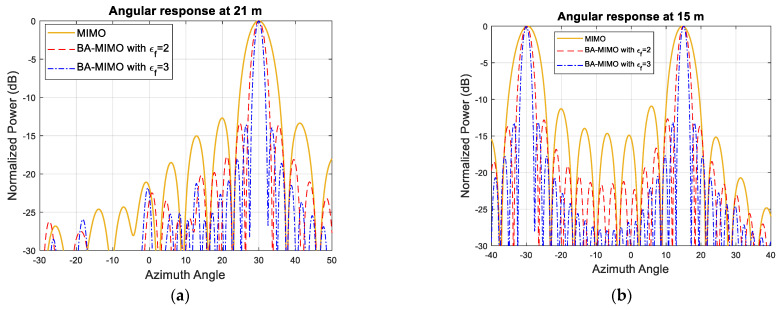
(**a**) The angular response of the MIMO antenna and Burg-aided MIMO antenna with $\varepsilon_{f}=2$ and $\varepsilon_{f}=3$ at a range of 21 m. (**b**) The angular response of the MIMO antenna and Burg-aided MIMO antenna with $\varepsilon_{f}=2$ and $\varepsilon_{f}=3$ at a range of 15 m.

**Figure 8:**
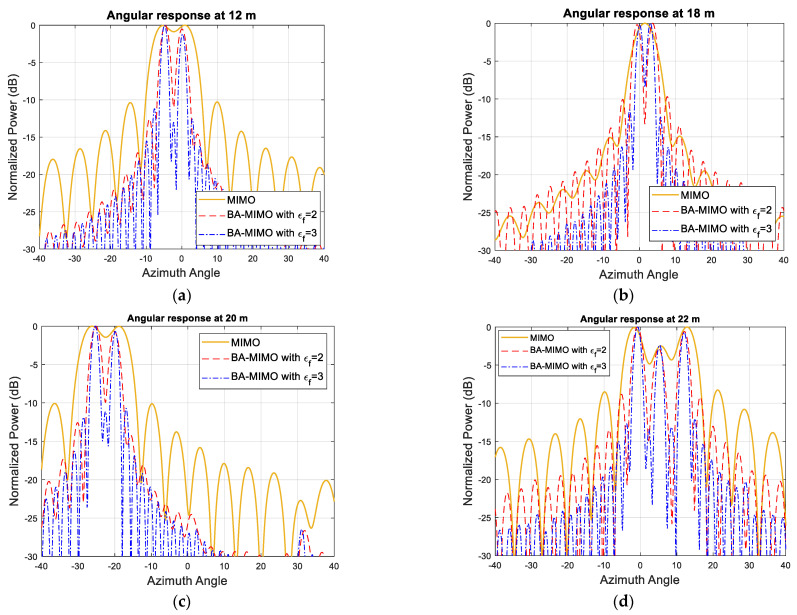
The angular response of the MIMO antenna and Burg-aided MIMO antenna with $\varepsilon_{f}=2$ and $\varepsilon_{f}=3$ at a range of (**a**) 12 m, (**b**) 18 m, (**c**) 20 m and (**d**) 22 m.

The simulated MIMO range–angle map for the scene in Figure 5a is demonstrated in Figure 5b. All closely positioned targets at a range of 12 m, 18 m, 20 m and 22 m are not resolved with this array resolution of 6°. However, extrapolating by a factor of two and three arrays with 36 and 54 equally spaced virtual elements refines the angular resolution by the same factor, so it becomes ~2.9° and 1.9°, respectively, and all targets are resolved as shown in Figure 6a–d. The range cuts corresponding to 21 m and 15 m are shown in Figure 7 to demonstrate improvement in terms of resolution refinement for a single target at 21 m and two targets at 15 m. The azimuth cuts of the relevant ranges which include closely positioned targets are demonstrated in Figure 8, and it is evident that the enhancement in angular resolution with Burg-aided MIMO applies not only to single or widely separated targets, but also to closely located targets that cannot be separated by the physical MIMO array.

These simulation results demonstrate that the BA is able to improve the angular resolution regardless of the number of targets and their positions. However, one should be cautious that when the extrapolation factor is more than three, the Burg algorithm may not work properly in all possible target positions due to the bias effect, as detailed in [17,38]. It should be noted that the far-field region may change after extrapolation, so the range assumed as far-field for the physical MIMO array may be near-field in the extrapolated array.

**Figure 9:**
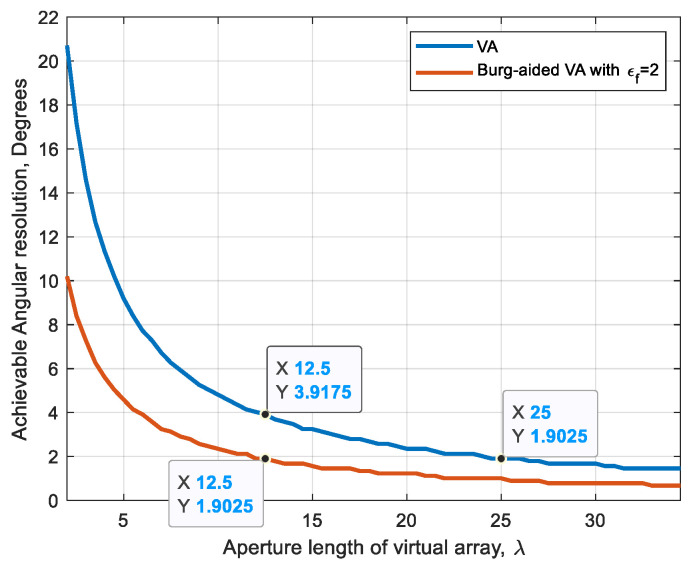
The achievable angular resolution versus the aperture length of the Burg-aided VA with $\varepsilon_{f}=2$ in comparison to the VA.

Figure 9 illustrates the Burg-aided resolution improvement by a factor of 2 in comparison with the VA.

### 5.2. Burg-Aided 2D MIMO

Let us assume that the required azimuth and elevation resolutions are $\theta_{Az}$ and $\theta_{El}$, respectively, requiring size of the aperture in both dimensions to be $l_{Az}$ and $l_{El}$, regardless of whether it is made by using a fully populated array or MMO virtual array. For instance, in the case of a conventional uniform L-shaped MIMO array, the dimension of the MIMO physical array size must be $\left(l_{Az}\times l_{El}\right)$.

In this section, several compact designs are considered, illustrating the advantages of the Burg-based approach. In particular, we show that equivalent angular resolutions can be achieved by using the 2D MIMO array of size $\left(\frac{l_{Az}+0.5\lambda }{4}\;\times \;\frac{l_{El}+0.5\lambda }{4}\right)$ with the first approach described in Section 2.2. It is worth stressing that the reduction in the physical aperture by almost a factor of 4 whilst ensuring the required performance can bring about dramatic advantages for applications where the miniature size of sensors is a requirement due to dense packaging within the infrastructure, as in the case of automotive radars.

As an illustrative example here, we consider the initial conventional L-shaped 20 Tx and 20 Rx MIMO array with λ/2 spacing giving a 5.2° angular resolution in both dimensions. Here, we show that a number of MIMO configurations with significant reductions in both (i) the number of physical elements and (ii) the physical size of the array can be designed to achieve the same resolution using the proposed approach.

**Figure 10:**
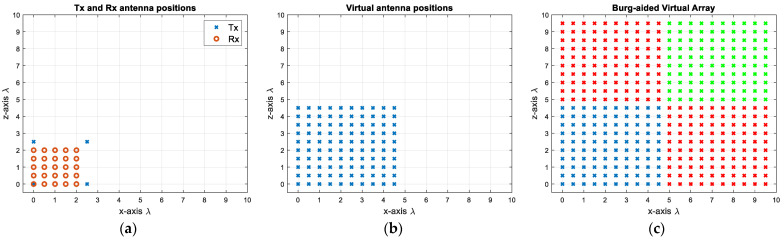
(**a**) The proposed 2D MIMO array with one single transmit/receive element, (**b**) the virtual array of the antenna configuration of (**a**,**c**) the extrapolated virtual elements via the BA (extrapolation along each dimension is shown in red, with further extrapolation shown in green).

**Figure 11:**
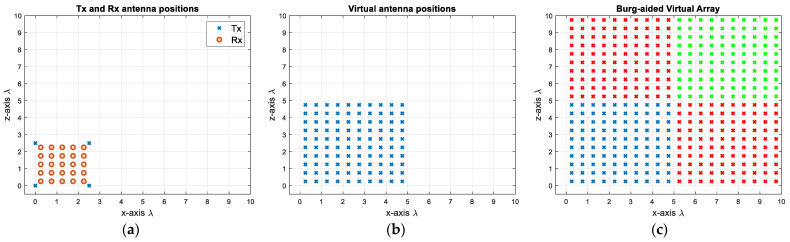
(**a**) Proposed MIMO with separated Tx and Rx. (**b**) VA of antenna configuration of (**a**). (**c**) Extrapolated virtual elements via BA.

**Figure 12:**
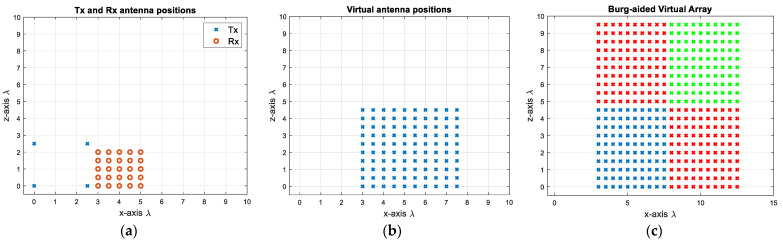
(**a**) When Tx and Rx packs are separated; (**b**) the VA of the antenna configuration of (**a**); (**c**) extrapolated virtual elements via the BA.

First, 4 Tx and 25 Rx are arranged in a compact design as shown in Figure 10a by determining the Rx positions via (9) and the Tx positions via (10). This decreases the number of antenna elements by 27.5%, but more importantly the radar response time in case of TDM is decreased dramatically by 80% due to fewer transmit elements. The corresponding virtual array includes 10 × 10 virtual elements with λ/2 spacing, as shown in Figure 10b. This gives roughly a 10.5° angular resolution in both dimensions. In order to attain a 5.2° beamwidth, the MIMO virtual aperture is extrapolated to twice its original size by using the BA. This is first applied to each row and each column of the virtual elements, and the extrapolated data are shown as red crosses in Figure 10c. Then, to populate the upper-right corner (shown by green crosses in Figure 10c), the previously extrapolated data—shown as red crosses—are used in a similar way, and the data of the upper-right corner elements are extrapolated by taking the mean of the new extrapolated data. In this way, the reconstructed virtual aperture resembles the virtual array configuration of the L-shaped MIMO of four times the size in each dimension with respect to the original physical aperture. However, as seen in Figure 10a, a Tx and a Rx are co-located at (0, 0) m, necessitating the use of a transceiver at this point. If this is not desired, the positions of the Rx antennas can be shifted by λ/4 in both the *x*- and *z*- axes, as shown in Figure 11a. Although it does not change the physical size of the 2D MIMO antenna, one could provide λ/4 spacing at high frequencies during the fabrication of such an antenna configuration. Another option could be to separate the Tx and Rx arrays as shown in Figure 12a, but this makes the MIMO array physically larger, yet still twice more compact than the conventional MIMO, with the advantages of easier manufacturing and avoiding coupling between elements. Each design would have 10 × 10 virtual elements with λ/2 spacing.

**Figure 13:**
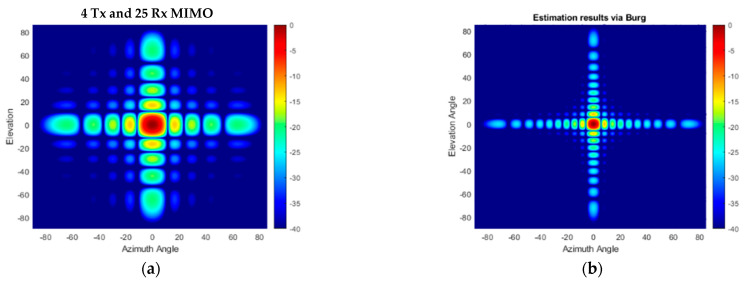
The angular response for a boresight target- (**a**) the compact size 2D MIMO; (**b**) the Burg-aided 2D MIMO.

**Figure 14:**
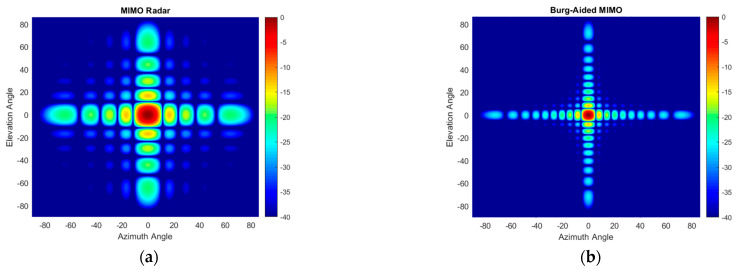
The angular response for a boresight target: (**a**) the antenna configuration in Figure 12a; (**b**) the Burg-aided 2D MIMO.

The angular response of the proposed compact-sized 2D MIMO antenna in Figure 10a and Figure 11a for a single target at a range of 15 m on boresight at (0°, 0°) is illustrated in Figure 13a and the Burg-aided extrapolation result is shown in Figure 13b. For the latter the angular resolutions in both dimensions are improved by a factor of approximately two, as expected, without degradation in the sidelobe level. The response of the configuration in Figure 12 to the same scenario with the single target is shown in Figure 14. It is seen that the change in the phase center of the array does not have an impact on the radar response. Therefore, the remaining analyses are performed by considering the MIMO antenna configuration in Figure 10a.

**Figure 15:**
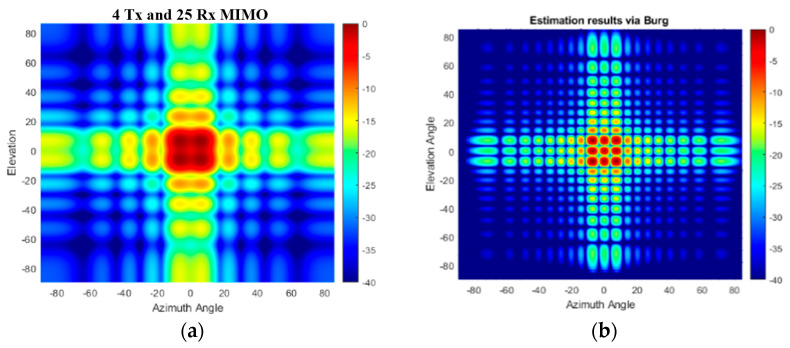
The angular response for multiple targets: (**a**) the compact-size 2D MIMO; (**b**) the Burg-aided 2D MIMO.

The performance of the proposed method in the case of multiple targets of the same radar cross section (RCS) positioned at the same range is also examined. An example of nine targets arranged within a (±6°, ±6°) area in azimuth and elevation angles is used in the simulation. Conventional beamforming with 4 Tx and 25 Rx physical elements of 2D MIMO does not allow us to resolve all targets shown in Figure 15a, while in the case of the Burg-aided MIMO, the targets are resolved without sacrificing the signal-to-background ratio, as illustrated in Figure 15b.

**Figure 16:**
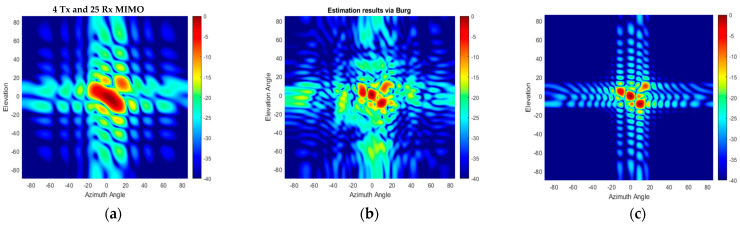
The angular response when targets have different RCSs: (**a**) the compact-size 2D MIMO, (**b**) the Burg-aided 2D MIMO and (**c**) the L-shaped 20 Tx by 20 Rx MIMO radar.

In the next multi-target scenario, the performance of the algorithm is examined when the targets have different RCSs. In this case, four targets are located at 15 m with [−8° 0° 5° 10°] in elevation and [10° 0° −10° 15°] in azimuth, respectively. The RCSs of the targets are [1 1 0.7 0.5] in normalized units, i.e., targets III and IV have 3 and 6 dB less reflectivity with respect to the first two targets. The Burg-aided MIMO correctly determines the positions and reflections of all four targets, as illustrated in Figure 16b, but has a slightly higher background level compared to the fully populated L-shaped 20 Tx by 20 Rx MIMO shown in Figure 16c.

**Figure 17:**
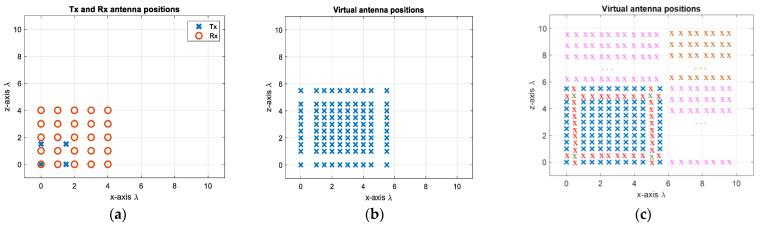
(**a**) The second proposed 2D MIMO array, (**b**) the virtual array of the antenna configuration of (**a**,**c**) the extrapolated virtual elements using the BA.

The second design approach from Section 2.2 has been used to come up with the configuration shown in Figure 17a. Whilst keeping the number of antenna elements the same as in the previous designs, the spacing between the transmitter elements is chosen to be 1.5λ, whereas the spacing between the receiver elements is λ, leading to a sparse virtual array (Figure 17b). In this design, to achieve the 5.2° angular resolution in both directions, initially the interpolation is carried out to populate the missing virtual array elements, followed by the extrapolation towards the right, upper, and upper-right directions so that a fully populated 20 by 20 extrapolated array is shown in Figure 17c. This proposed 2D MIMO antenna configuration can be particularly useful when higher frequencies, potentially in the sub-THz region with mm order wavelengths [39,40], are desired. In the proposed MIMO with one wavelength as the minimum spacing, fabrication will be less challenging.

**Figure 18:**
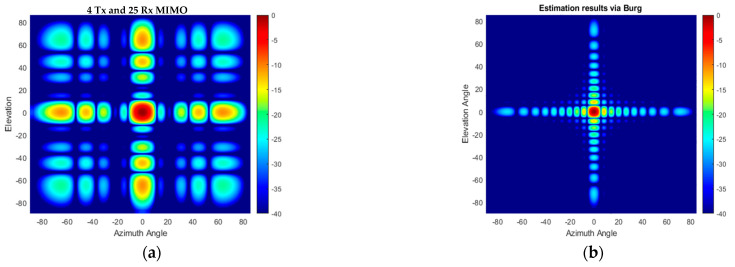
The angular response for a boresight target: (**a**) the second proposed 2D MIMO and (**b**) after the BA is applied to the second configuration.

**Figure 19:**
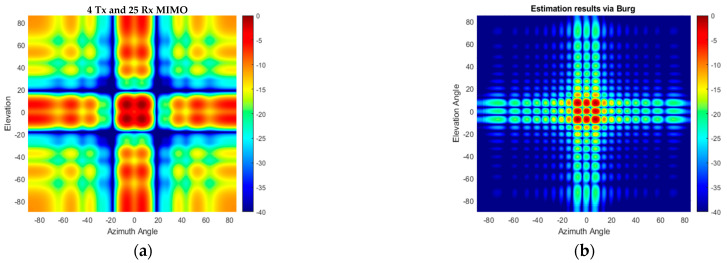
The angular response for the multi-target scenario: (**a**) the second proposed 2D MIMO; (**b**) after the BA is applied to the second configuration.

Regarding the performance of the BA in such antenna configurations, the angular responses of the original VA and Burg-aided MIMO in the case of single-target and multi-target scenarios are illustrated in Figure 18 and Figure 19, respectively. Due to the sparsity in the virtual array of the 2D MIMO, the angular responses contain high sidelobes, but after applying the proposed method, the response improves dramatically due to interpolation of the thinned array, as well as ensuring the desired angular resolutions.

It is worth stressing here that this specific design of array where Burg-aided interpolation and extrapolation are expected is driven by intended application requirements and the limitations of fabrication, but there are no fundamental limitations of the approach to improving resolution in 2D MIMO.

## 6. Experimental Setup and Results

For experimental verification of the proposed technique, the INRAS Radarbook MIMO radar operating at 77 GHz has been used with a 2 GHz bandwidth, giving a range resolution of 7.5 cm. The INRAS Radarbook frontend is a 1D MIMO with four transmitters and eight receivers aligned in azimuth, giving thirty-two virtual linear elements [41]. Three of the elements overlap, resulting in a twenty-nine-element linear array with half-wavelength spacing.

**Figure 20:**
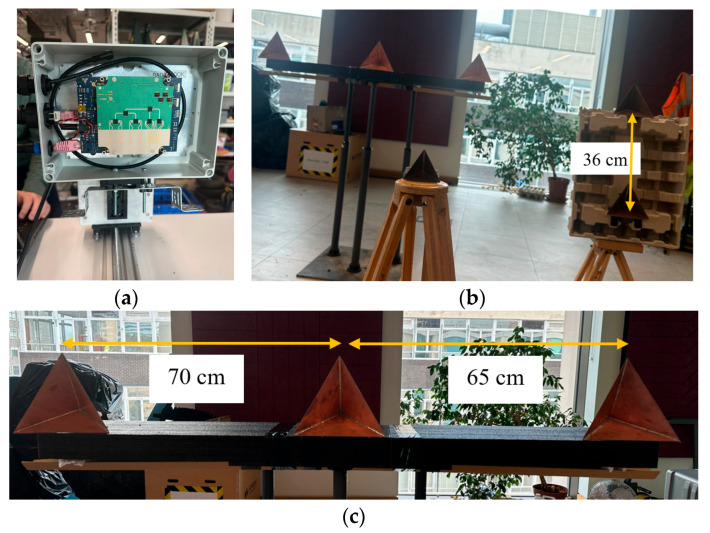
(**a**) INRAS MIMO radar with 77 GHz frontend, (**b**) photograph from experimental site and (**c**) three corner reflectors at 3.7 m.

The experimental setup in the Microwave Systems Laboratory of the School of Engineering at the University of Birmingham is shown in Figure 20. Six triangular corner reflectors have been used as point targets of known RCSs during the experiment. The corner reflector with 7 cm edges of an 8.21 dBsm RCS has been placed at a 1.7 m range at (0°, 0°) with respect to the phase center of the antenna, as shown in Figure 20b. Two corner reflectors with edges of 10 cm of a 14.41 dBsm RCS were placed at a range of 2.5 m at 15° in azimuth with a difference of 0.36 m in elevation. The other three corner reflectors with a 15 cm edge (RCS of 21.45 dBsm) were positioned at ranges of 3.7 m and 0.25 m in elevation with distances between them of 0.7 m and 0.65 m, respectively, in azimuth, as shown in Figure 20c. The corner reflector on the right-hand side is on the boresight of the radar antenna.

**Figure 21:**
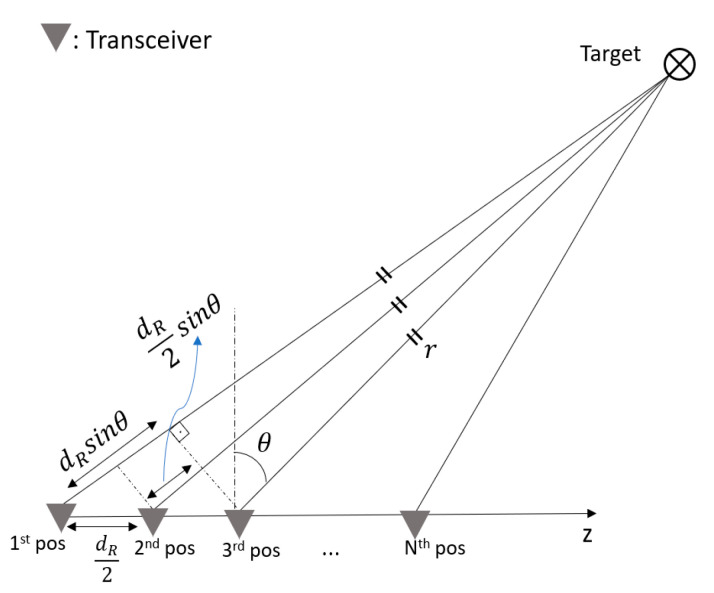
The geometrical explanation of the direct path of the signal at each transceiver’s position.

For 3D scene mapping in range, azimuth and elevation, the third dimension was added through consecutive changes in the position of the 1D MIMO radar in elevation using an 8MT175 Standa vertical positioner with minimum displacement steps of 2.5 μm [42]. The geometry used for the data collection process is shown in Figure 21. Considering a monostatic radar configuration, while the radar is in the first position, the distance between the transceiver and target is ${r+d}_{R}sin\theta$, so the total signal travelling distance is ${2r+2d}_{R}sin\theta .$ When the transceiver is moved to the second position by $d_{R}/2$ towards the z-axis, the total distance becomes ${2r+d}_{R}sin\theta .$ It is therefore clear that a $d_{R}/2$ movement of the transceiver results in a $d_{R}$ path difference between data collected in each consecutive position.

In the experiment, to collect the 3D data using the 1D MIMO radar, the position of the radar was moved each time by λ/4 in elevation, so that the spacing between the virtual elements in elevation was λ/2 as both transmitters and receivers were moved simultaneously in this method. For experimental validation of the proposed method with the first 2D MIMO antenna configuration given in Section 5.2 as an example, the radar was moved in elevation 20 times, and the 3D data cube of size ($N_{range},\;29,\;20$) was formed, where $N_{range}$ is the number of range bins, and 29 and 20 are the numbers of the array elements in the azimuth and elevation dimensions, respectively. To achieve a virtual array of an L-shaped 20 × 20 MIMO configuration, the data of the first 20 elements of the 1D MIMO in azimuth were used, and data collected from 21 to 29 virtual elements on the 1D MIMO azimuth were discarded. Hence, a fully populated 20-by-20 virtual array was obtained and its radar response serves as an experimental reference response for our proposed method.

To compare the proposed method and the L-shaped 20 × 20 2D MIMO experimental responses, the data cube was reduced from ($N_{range},\;20,\;20$) to ($N_{range},\;10,\;10$), which corresponds the virtual array configuration in Figure 10b, and the remaining data were estimated by applying the BA as in Section 4.

**Figure 22:**
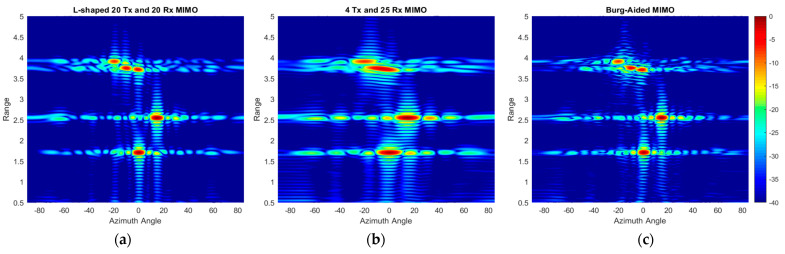
Range–azimuth angle responses of (**a**) L-shaped 20 × 20 MIMO radar, (**b**) compact-size 2D MIMO radar with 4 Tx and 25 Rx and (**c**) with Burg estimation applied to compact-size 2D MIMO.

**Figure 23:**
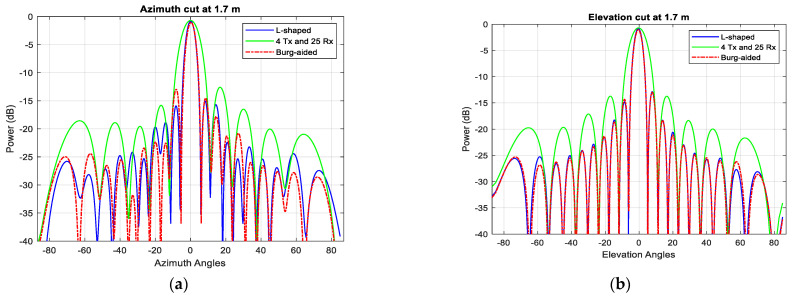
The responses of the L-shaped MIMO, compact-size MIMO and Burg-aided MIMO for (**a**) an azimuth cut of the corner reflector on the boresight at 1.7 m and (**b**) an elevation cut of the corner reflector on the boresight at 1.7 m.

Figure 22 shows the range–azimuth map of (a) the L-shaped 20 × 20 MIMO, (b) the 4 Tx and 25 Rx 2D MIMO, and (c) the extrapolated virtual array of the 4 Tx and 25 Rx 2D MIMO using the BA without the use of any averaging or weightings. The azimuth response of the compact-sized 2D MIMO shown in Figure 22b is wider because there are 10 virtual elements in each row, and hence it cannot resolve the corner reflectors at a range of 3.7 m. However, the Burg-aided MIMO estimation enhances the azimuth response and obtains a range–azimuth response very close to that of the L- shaped full MIMO, as seen in Figure 22c. To highlight the improvement in angular resolutions in the Burg-aided MIMO, both zero-azimuth and zero-elevation cuts of the experimental response from the corner reflector at 1.7 m are shown in Figure 23a,b. Although the elevation response is a sinc function, the azimuth response exhibits some irregularities in the sidelobes, which is potentially caused by the multipath reflections in the lab as the INRAS Radarbook has a wider beam in azimuth of 51° [41].

**Figure 24:**
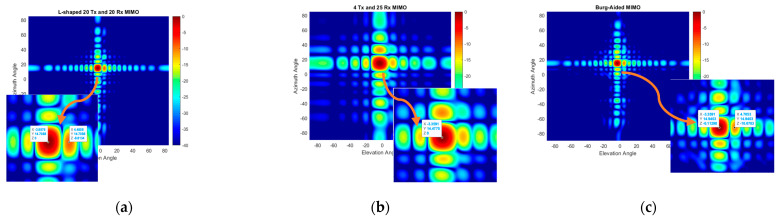
2D MIMO azimuth–elevation angle maps of two corner reflectors at 2.5 m: (**a**) L-shaped 20 × 20 MIMO radar, (**b**) compact-size 2D MIMO radar and (**c**) Burg-aided MIMO.

The azimuth–elevation cut of 3D data at a range of 2.5 m is shown in Figure 24. Whilst the compact-size 2D MIMO can detect the two corner reflectors as just one target, as seen in Figure 24b, the Burg-aided MIMO separates the targets, as expected, which is seen in Figure 24c. The corner reflector at roughly a −4° elevation angle has slightly higher reflection, compared to the reflector at a around 4° elevation angle, although they are identical. The reason could be slight orientation differences of the corner reflectors, or the cardboard apparatus used to mount the corner reflector.

In Table 1, the sensitivity of the proposed method (the Burg-aided 2D MIMO) to position precision and power accuracy, compared to a reference MIMO array (L-shaped 20 Tx and 20 Rx 2D MIMO), is evaluated using the measurement results. It is apparent that the difference in the obtained power values is not higher than ±1.3 dB, whereas the azimuth and elevation estimations differ by no more than ±0.5°, which is within 5% of the boresight beamwidth of the actual, non-extrapolated MIMO. Moreover, the Burg-aided MIMO gives values closer to that of the ground truth. Given that both azimuth and elevation angular resolutions of a compact-sized 2D MIMO are enhanced by a factor of two via the proposed method, regardless of the number of targets, their RCS values and their positions can be determined, and these accuracies agree very well with some discrepancy corresponding to the expected measurement error.

## 7. Conclusions

The main benefit of using the extrapolation offered by the BA is the opportunity to obtain very fine resolution by using very compact hardware with a small number of physical elements and a small occupied area. This would offer not only a cost-effective solution but allow for the use of such sensors in applications where the allocation of space is under severe constrains, as in the case of automotive infrastructure. Moreover, fewer transmit elements in the TDM MIMO array would mean larger and therefore more favorable unambiguous Doppler, which would make a radar better suited for detection from a moving platform and with moving targets. In this paper, the performance of the Burg algorithm in interpolating and extrapolating data for improved spatial resolution as well as the associated limitations have been examined in both 1D and 2D MIMO arrays. Also, in the experiment, the authors have described the methodology used to generate 3D data (range, azimuth and elevation information) using a 1D MIMO array.

The general rule of how to design an initial compact MIMO allowing for extrapolation in cases of both regular and sparse element positioning has been presented and discussed with several design realizations. It has been shown that even an extrapolation factor of 3 can deliver expected super-resolution performance, though higher factors as well as transition to the near-field zone of the larger virtual antenna put constraints on the viability of higher factors. The proposed method has been validated through both simulation and experimentation at 77 GHz with targets of different RCSs.

Importantly, while point-like targets have been considered to validate the simulation results, the value of this approach is that in the case of extended targets, the Burg-aided MIMO resolution would define more image-like extended target representation within the radar map, thanks to the sensitivity of mm wave radar to surface texture. Based on this, in our next follow-on paper, we will demonstrate the performance of the Burg algorithm in conjunction with several beamforming approaches to produce enhanced imagery of road environments using off-the-shelf automotive MIMO radar from moving vehicles.

## Figures and Tables

**Table 1:** Comparison of L-Shaped MIMO and Burg-aided MIMO in terms of power and positional accuracy of targets.

		L-Shaped 20 Tx and 20 Rx MIMO			Burg-Aided MIMO			Changes		
Target	Range	Power (P_L_)	Azimuth (Az_L_)	Elevation (El_L_)	Power (P_B_)	Azimuth (Az_B_)	Elevation (El_B_)	ΔP = P_L_ − P_B_	ΔAz = Az_L_ − Az_B_	ΔEl = El_L_ − El_B_
**Target I**	1.7 m	−0.81 dB	0°	−0.448°	−1.063 dB	0°	−0.448°	0.253 dB	0°	0°
**Target II**	2.5 m	0 dB	14.709°	−3.808°	−0.113 dB	14.940°	−3.359°	0.113 dB	−0.231°	−0.449°
**Target III**	2.5 m	−8.813 dB	14.709°	4.481°	−10.078 dB	14.940°	4.705°	1.265 dB	−0.231°	−0.224°
**Target IV**	3.711 m	−4.90 dB	−0.671°	2.911°	−4.078 dB	−0.895°	2.911°	−0.822 dB	0.224°	0°
**Target V**	3.743 m	−4.630 dB	−9.897°	3.359°	−4.052 dB	−9.670°	3.135°	−0.578 dB	−0.227°	0.224°
**Target VI**	3.903 m	−6.808 dB	−19.867°	2.687°	−7.047 dB	−20.106°	2.686°	0.029 dB	0.239°	0.01°
